# A Study of Drug Utilization Pattern and Pharmacoeconomic Analysis of Immunosuppressant Drugs in Patients With Skin Disorders in a Tertiary Care Hospital in Bihar

**DOI:** 10.7759/cureus.48541

**Published:** 2023-11-08

**Authors:** Adil A Shakur, Raushan K Ranjan, Rajesh Sinha, Saajid Hameed, Lalit Mohan

**Affiliations:** 1 Department of Pharmacology, Indira Gandhi Institute of Medical Sciences, Patna, IND; 2 Department of Skin and Venereal Diseases, Indira Gandhi Institute of Medical Sciences, Patna, IND

**Keywords:** skin disorders, pharmacoeconomic, immunosuppressant, drug-utilization, cost-effectiveness, corticosteroids

## Abstract

Aims

The cost-effective therapy of immunosuppressant drugs in dermatological conditions will not only lead to adherence to rational prescribing but will also increase patient compliance with fewer dropouts due to cost factor. Thus, this study was done to determine and compare the drug utilization pattern, prescribed daily dose/defined daily dose (PDD/DDD) defined by WHO, and the cost-effectiveness ratio of different immunosuppressants.

Methods and material

Prescriptions for patients with skin disorders prescribed with any one systematic or topical immunosuppressant were collected. The utilization of drugs in different skin disorders was expressed as frequency and percentage. PDD was compared with DDD as per the WHOCC-Anatomical Therapeutic Chemical (ATC)/DDD index. The pharmacoeconomic analysis was done using a cost-effectiveness ratio.

Statistical analysis

Descriptive statistics were used to calculate percentages, frequency, and 95% CI. The cost-effectiveness ratio in terms of SFDs (symptom-free days) was defined as the total cost of the initial antibiotic during the study period divided by the number of SFDs (cost/ SFD) and was expressed as mean±standard deviation, and the Kruskal-Wallis test was used to determine statistical significance of difference.

Results

Immunosuppressants were prescribed in 117 (19.12%) prescriptions out of a total of 612 prescriptions. Deflazacort was the most utilized systemic immunosuppressant prescribed in 27.18% of cases and was commonly prescribed for irritant contact dermatitis (ICD) and allergic contact dermatitis (ACD) followed by prednisolone and betamethasone. Tacrolimus was the most utilized topical immunosuppressant prescribed in 15.90% of patients and was commonly used for ICD and vitiligo followed by clobetasol and mometasone. Betamethasone, prednisolone, clobetasol, and mometasone had better cost-effectiveness. PDD/DDD of all immunosuppressants was less than one except prednisolone, which had a PDD/DDD ratio of 3.52.

Conclusions

The cost-effectiveness of steroids has the advantage of providing better patients’ adherence to pharmacotherapy, but over-prescribing could also lead to long-term adverse effects of steroids. Pharmacovigilance research should also incorporate pharmacoeconomic analysis to determine the relation between these two aspects.

## Introduction

Skin, the biggest organ in human beings, is a component of the integumentary system and, hence, is vulnerable to injury from both extrinsic and intrinsic causes including genetic, metabolic, and immune responses as well as microbes or environmental and chemical agents. Accordingly, a lot of systemic disorders can be recognized by their dermatological symptoms, and therefore, it is referred to symbolically as a mirror for different internal ailments [1,2].

The majority of skin disorders are chronic and need a longer period of therapy; thus, they have a substantial negative impact on the community in terms of quality of life by raising physical, social, and mental discomfort as well as causing economic hardship [3]. Psoriasis, contact dermatitis, autoimmune bullous diseases (AIBDs), and alopecia areata (AA) are a few examples of inflammatory skin conditions that have a significant negative impact on quality of life and create a significant economic burden.

Different pathological consequences involving proinflammatory immune cells and structural cells of tissue result from the deregulation of the cutaneous immune system. These then turn into biological targets for the synthesis of immunosuppressants, such as biological drugs that target specific extra-cellular or cell-surface receptors. So, the use of immunomodulators is critical in the pharmacotherapy of various skin disorders [4]. The traditional approach to treating these skin disorders has concentrated on managing symptoms with topical and systemic corticosteroids and other systemic immunosuppressive agents such as antimetabolites, alkylating agents, cyclosporine, and mycophenolate mofetil [4]. Due to the absence of specific targets, they completely suppress the immune system apart from the control of the exaggerated immune responses to antigens, which occasionally results in major adverse events in addition to unfavorable side effects [5]. It is possible to use these medications effectively and wisely for skin disorders if one is familiar with the efficacy in clinical trials, cost-effectiveness, adverse effect profile, and dose for various conditions [6].

The cost-effective therapy of immunosuppressant drugs in dermatological condition will not only lead to adherence to rational prescribing but will also increase the patient compliance with fewer dropouts due to cost factor. On the other hand, overuse or misuse of drugs can lead to a greater incidence of adverse drug reactions and chronic adverse effects that could lead to over-reporting of the cost-effective drug having poor safety [7]. This adversity can be countered with a distinct field of study in health economics called pharmacoeconomics. It is the analysis and comparison of two or more forms of pharmacotherapy that provide an estimate of the cost and effect in terms of effectiveness and quality of life [8]. These studies offer a roadmap for ensuring that scarce resources are utilized skillfully and scientifically to maximize healthcare facilities, especially in an underdeveloped nation [9]. This intent can be fulfilled by detecting the underuse and overuse of drugs and evaluating their cost-effectiveness, which is crucial in the identification of factors leading to adverse effects and poor patient’ compliance with pharmacotherapy. Hence, this study was planned to evaluate drug utilization patterns and generate pharmacoeconomic data on immunosuppressant drugs in patients with skin disorders in a tertiary care hospital in eastern India. The objective was to determine and compare the drug utilization pattern, prescribed daily dose/defined daily dose (PDD/DDD) defined by the World Health Organization (WHO), and the cost-effectiveness ratio of different immunosuppressants.

## Materials and methods

This was an observational and analytical study conducted in the outpatient department of skin and venereal disease in a tertiary care hospital in eastern India. The study was conducted for the duration of six months (October 2022 - April 2023) after getting approval from the institutional ethics committee (232/IEC/IGIMS) under the principles of the Declaration of Helsinki and good clinical practice.

Inclusion criteria

Patients of either gender aged between 18 and 60 years visiting the outpatient department of skin and venereal disease for the first time with the particular diagnosis and prescribed with at least one systemic and topical immunosuppressant were included in our study.

Exclusion criteria

Prescription of follow-up patients having an active infection diagnosed clinically or microbiologically or having any immunodeficiency disorders or having low neutrophil or lymphocyte count or having cardiovascular, renal, or hepatic disorder were excluded. Pregnant and lactating women were also excluded from our study.

Sampling method

Consecutive sampling method was used and all the prescriptions of patients with skin disorders found eligible for study as per inclusion and exclusion criteria were collected.

A total of 612 prescriptions were collected during the study period of which immunosuppressant was prescribed in 117 prescriptions. This was in accordance with the WHO recommendation of including at least 600 encounters in a cross-sectional survey to assess present prescribing practices [10]. The prescriptions were assessed by using the WHO core drug prescribing indicators, which include the mean number of drugs prescribed per prescription, the proportion of generic drugs prescribed, the percentage of prescriptions with antibiotics, the percentage of prescriptions with injections, and the percentage of drugs prescribed from the list of essential medicine or local formulary. The National List of Essential Medicines 2003 of India was used to assess the number of drugs prescribed from the essential list [11]. The prescribed immunosuppressant drugs were categorized in accordance with the Anatomical Therapeutic Chemical (ATC) Classification. The PDD was calculated by getting the mean of the daily doses of immunosuppressant drugs. DDD of a particular immunosuppressant was found in the WHOCC ATC/DDD index. Thereafter, the PDD to DDD ratio of the immunosuppressant was calculated [12]. The cost-effectiveness ratio in terms of SFDs (symptom free days) was defined as the total cost of an immunosuppressant in one month divided by the number of SFDs per month (Cost/ SFD) [13]. The cost of the drug was taken from the available branded/generic forms of the prescribed drug from the central pharmacy of the hospital. The number of tablets/pills taken from the patient for a duration of one month was multiplied by the cost of each unit. The cost of topical immunosuppressants was calculated by the amount of drug consumed (in mL) divided by the total amount of the unit and then multiplied by the cost of the unit.

Statistical analysis

Data collected on all parameters from the prescriptions were presented in tabular form using Microsoft Excel 365 (Microsoft Corporation, Redmond, Washington, United States) and then transferred to SPSS version 24 ((IBM Corp., Armonk, NY) for further statistical analysis. Frequency and percentage with a 95% confidence interval (CI) of the utilization of different drugs in various dermatological diseases were calculated using descriptive statistics. The cost-effectiveness ratio in terms of cost/SFD was expressed as mean±standard deviation, and the Kruskal-Wallis test was used to determine the statistical significance of the difference between various immunosuppressants with a p-value of less than 0.05 as the measure of statistical significance.

## Results

A total of 612 prescriptions of new patients attending the outpatient department of skin and venereal disease of a tertiary care hospital in eastern India were collected of which 117 (19.12%) patients were prescribed immunosuppressants. The average number of drugs per prescription was found to be 3.9. Polypharmacy was observed in 14 prescriptions (11.96%). Only 8.58% of drugs were prescribed by generic name. Antibiotics were prescribed in 63 cases (10.29%), injectables were prescribed in only 29 cases (4.74%) and more than 70% of drugs prescribed were from the national list of essential medicines.

The number of male patients was 54 (46.15%) and the number of female patients was 63 (53.85%) with a male-to-female ratio of 6:7. Most of the patients belonged to the age group of 31-40 years with a mean age of 34.68±7.86.

Out of 117 prescriptions containing immunosuppressants, the most common indication was irritant contact dermatitis (ICD) (n=25, 21.37%) followed by allergic contact dermatitis (ACD) (n=22, 18.80%), psoriasis, and vitiligo (n=19, 16.24%). Skin disorder for which immunosuppressants were prescribed is given in Table 1. PDD/DDD of all immunosuppressant was less than one except prednisolone which had PDD/DDD ratio of 3.52.

**Table 1:** Skin disorders associated with utilization of immunosuppressants The data has been represented as n, %, and 95% CI. CI, confidence interval; SLE, systemic lupus erythematosus; SJS, Stevens Johnson syndrome; DLE, discoid lupus erythematosus

Disease	Number of prescriptions with immunosuppressant	Percentage of prescription (95 % CI) n=117
Irritant contact dermatitis	25	21.37 (14.91 to 29.64)
Allergic contact dermatitis	22	18.80 (12.76 to 26.83)
Psoriasis	19	16.24 (10.65 to 23.98)
Vitiligo	11	9.4 (5.33 to 16.05)
Lichen planus	9	7.69 (4.1 to 13.97)
Lichen simplex chronicus	8	6.84 (3.51 to 12.91)
Alopecia areata	6	5.13 (2.37 to 10.74)
DLE	6	5.13 (2.37 to 10.74)
Chronic urticaria	4	3.42 (1.34 to 8.46)
Dermatomyositis	3	2.56 (0.7 to 7.27)
SLE	3	2.56 (0.7 to 7.27)
SJS	1	0.85 (0.04 to 4.68)

A total of 195 immunosuppressants were prescribed in 117 prescriptions. Out of which, the drug deflazacort was the most utilized immunosuppressant prescribed in 27.18% (n=53) of cases and was commonly prescribed for ICD and ACD. Prednisolone was prescribed in 16.41% (n=32) of patients and was used commonly in ICD, ACD, vitiligo, psoriasis, lichen planus, and discoid lupus erythematosus (DLE). Tacrolimus was the most utilized topical immunosuppressant prescribed in 15.90% (n = 31) of patients and was commonly used in ICD and vitiligo (Table 2).

**Table 2:** Frequency of utilization of different immunosuppressants The data has been represented as n, %, and 95% CI. CI, confidence interval; ICD, irritant contact dermatitis; ACD, allergic contact dermatitis; LSC, lichen simplex chronicus; DLE, discoid lupus erythematosus; SJS, Steven Johnson syndrome; SLE, systemic lupus erythematosus

Immunosuppressant drug	Frequency	Percentage of total utilization (95% CI)	Disease involved (n)
Tablet deflazacort	53	27.18 (21.42 to 33.82)	ICD (18), ACD (17), LSC (7), psoriasis (6), chronic urticaria (4), DLE (1)
Tablet prednisolone	32	16.41 (11.87 to 22.25)	ICD (7), ACD (5), vitiligo (5), psoriasis (4), lichen planus (4), DLE (3), lichen simplex chronicus (1), alopecia areata (2), SJS (1)
Topical tacrolimus	31	15.90 (11.43 to 21.68)	ICD (14), vitiligo (11), lichen planus (5), SJS (1)
Topical mometasone	22	11.28 (7.57 to 16.49)	ICD (6), LSC (5), ACD (6), psoriasis (4), lichen planus (3)
Tablet betamethasone	15	7.69 (4.72 to 12.3)	Vitiligo (6), lichen planus (5), alopecia areata (4)
Topical clobetasol	14	7.17 (4.32 to 11.69)	LSC (6), psoriasis (4), ACD (4)
Topical fluocinolone acetonide	9	4.62 (2.45 to 8.54)	Alopecia areata (6), DLE (3)
Tablet cyclosporine	8	4.1 (2.09 to 7.89)	Psoriasis (8)
Tablet methotrexate	5	2.56 (1.1 to 5.86)	Dermatomyositis (2), Psoriasis (1), SLE (1), DLE (1)
Tablet azathioprine	4	2.05 (0.80-5.16)	Dermatomyositis (1), SLE (2), DLE (1)
Topical fluticasone propionate	2	1.03 (0.18 to 3.66)	DLE (2)

Cyclosporine was used in eight patients diagnosed with psoriasis whereas methotrexate and azathioprine were commonly used in dermatomyositis and systemic lupus erythematosus. Utilization of immunosuppressants with their indication is given in Table 2.

**Figure 1:**
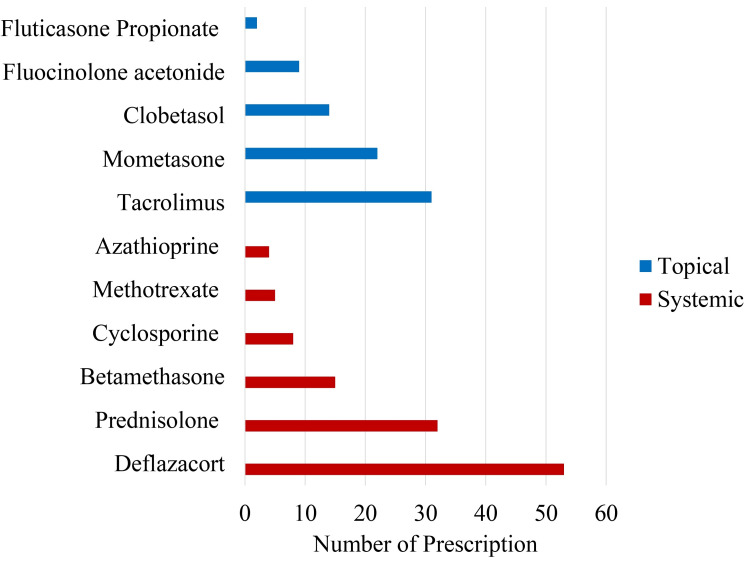
Frequency of utilization of immunosuppressant drugs The data has been represented as n.

Utilization of systemic immunosuppressants was greater than topical Immunosuppressants, and steroids were used more frequently than other immunosuppressants as shown in Figure 1.

Among systemic immunosuppressants, betamethasone was found to be the best cost-effective drug (p<0.0001) in terms of cost-effectiveness ratio followed by methotrexate. Azathioprine was found to have the poorest cost-effectiveness ratio of 51.26 (Table 3).

**Table 3:** Comparison of cost-effectiveness in terms of cost/SFD of different systemic immunosuppressants The data has been represented as mean±SD. P-value (Kruskal-Wallis test) <0.0001: significant. SFD, symptom-free days; SD, standard deviation

Immunosuppressant drug	Cost in rupees/SFD per month (mean±SD)
Deflazacort	18.29±4.39
Prednisolone	3.58±0.76
Betamethasone	1.52±0.23
Cyclosporine	26.26±8.16
Methotrexate	2.83±0.06
Azathioprine	51.26±2.57

Among topical immunosuppressants, clobetasol had the best cost-effectiveness ratio followed by mometasone (p<0.0001). Fluticasone propionate was found to have poorest cost-effectiveness ratio (Table 4).

**Table 4:** Comparison of cost-effectiveness in terms of cost/SFD of different topical immunosuppressants The data has been represented as mean±SD. P-value (Kruskal-Wallis test) <0.0001: significant. SFD, symptom-free days; SD, standard deviation

Immunosuppressant drug	Cost in rupees/SFD per month (mean ± SD)
Tacrolimus	20.44±5.67
Mometasone	13.80±2.48
Clobetasol	8.14±1.59
Fluocinolone acetonide	15.48±3.91
Fluticasone propionate	22.68±5.83

**Figure 2:**
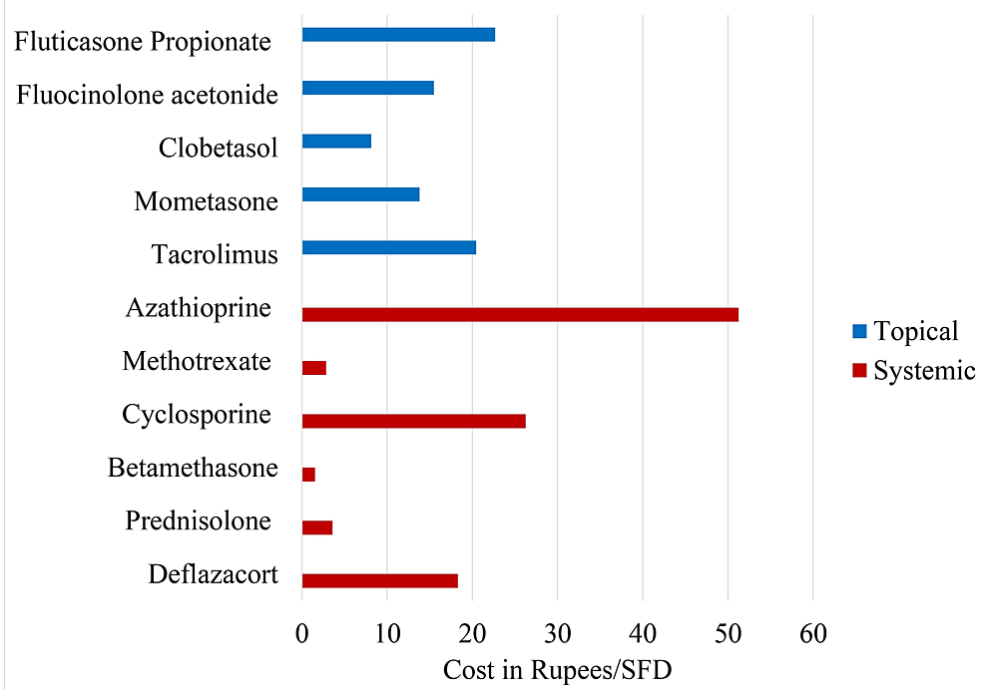
Comparison of cost-effectiveness in terms of cost/SFD of immunosuppressant drugs The data has been represented as n. SFD, symptom free days

By comparing Figure 1 and Figure 2 (as shown below), we can see that more frequently utilized immunosuppressants have low cost/SFD (better cost-effectiveness) and less frequently utilized drugs have poor cost-effectiveness (high cost/SFD).

PDD/DDD of all immunosuppressant was less than one except prednisolone, which had PDD/DDD ratio of 3.52 (Table 5).

**Table 5:** Comparison of PDD/DDD of different systemic immunosuppressants The data has been represented as a ratio expressed in its decimal form. ATC, Anatomical Therapeutic Chemical; PDD, prescribed daily dose; DDD, defined daily dose

Immunosuppressant drug	ATC code	PDD/DDD ratio
Deflazacort	H02AB13	0.60
Prednisolone	H02AB06	3.52
Betamethasone	H02AB01	0.67
Cyclosporine	L04AD01	0.80
Methotrexate	L04AX03	0.86
Azathioprine	L04AX01	0.93

## Discussion

Our skin serves as a barrier against pathogen invasion, but it is also a site where sterile inflammatory processes, such as tumor immunity, allergies, and autoimmune reactions, may contribute to diseases. In this study, about 20% of prescriptions surveyed in the department of skin and venereal disease of a tertiary care hospital in eastern India indicate that over-activity of the immune pathway is a common pathogenesis of many skin disorders. Immunocompromised individuals receiving immunosuppressive therapy after organ transplants have impaired immunity to malignant cells, yet this immunity becomes adopted when different skin malignancies and warts are treated with imiquimod. Our skin when exposed to harmless external substances, allergic reactions the presumably develop to protect against parasite invasion, can cause diseases such as ACD. Vitiligo, systemic lupus erythematosus, psoriasis, and other skin disorders are caused by autoimmune pathways, which may be brought on by misdirected anti-pathogen or anti-tumor immune processes [14].

The stromal cells such as keratinocytes, endothelial cells, fibroblasts, and adipocytes, along with immune cells that originate from the bone marrow such as dendritic cells, monocytes, natural killer cells, mast cells, and T lymphocytes must function in a coordinated manner in order for the skin to function properly and for fulfilling their roles. Among the cells derived from bone marrow that are discovered in the skin, a few are collections of resident cells that move to the skin where they terminally differentiate and predominantly are located there, some re-circulate constantly and execute a monitoring function, some are recruited to combat infective agents for a brief period of time, and others are recruited and maintained as memory cells to guard against potential reinvasion [14].

In this study, polypharmacy was found to be present in only 11.96% (n=14) of prescriptions as most of the patients belonged to the age group of 31-40 years, and polypharmacy is more common in the elderly [15]. Only 8.58% (n=10) of drugs were prescribed by generic name. This may be probably due to substandard quality and low effectiveness of generic drugs in our country [16-18]. Generic forms of the prescribed immunosuppressants were cheaper than the branded form, but we have determined the cost of the therapy from the price of the branded/generic name from the central pharmacy of the hospital. Antibiotics were prescribed in only 10.29% (n=12) of prescriptions, which was lower than the findings of Yuwante et al. and Pathak et al. [19,20]. Most of the immunosuppressants were found in the national list of essential medicines, which indicates that the government of India has done remarkable work in ensuring drug availability for common diseases [21]. Females were found to be slightly more frequently associated with skin diseases requiring immunosuppressants compared to males, which is supported by the findings of Anderson et al. [22].

Among the drugs, deflazacort was the most utilized immunosuppressant prescribed in 27.18% (n=53) of cases and was commonly prescribed for ICD and ACD. Prednisolone was prescribed in 16.41% (n=32) of patients and was used commonly in ICD, ACD, vitiligo, psoriasis, lichen planus, and discoid lupus erythematosus. However, the PDD/DDD ratio of prednisolone was 3.52, which means that it was given in higher doses than recommended. Surprisingly, prednisolone was cheaper than deflazacort, but its utilization was lower than deflazacort as per the number of prescriptions. In various circumstances, the bioequivalence of deflazacort and prednisolone has been examined. Based on phytohemagglutinin-induced T lymphocyte proliferation in in vitro experiments, 15 mg of deflazacort reduces T lymphocyte reactivity in normal persons to the same level as 12.5 mg of prednisolone, but for a greater duration [23]. Avioli et al. calculated the potency ratio of deflazacort versus prednisolone to be 1.28 according to the findings of seven studies of various designs, comprising a double-blind cross-over trial, paired patient studies, and between-patient studies including 160 participants [24]. Deflazacort generally seems to have a lesser influence than prednisone on variables that could be linked to the onset of steroid-induced osteoporosis. Additionally, deflazacort seems to have a less detrimental effect on the growth rates of bones in children who have conditions requiring corticosteroid therapy. Deflazacort might, therefore, have less severe metabolic side effects than prednisone, but more thorough long-term studies are needed to prove this. It appears to have less adverse effects on the density of bones than prednisone when administered at doses approximately comparable to anti-inflammatory efficacy, according to histomorphometry and densitometry studies [25]. Tacrolimus was the most frequently prescribed topical immunosuppressant, which was most prescribed for vitiligo. The effectiveness of tacrolimus in vitiligo has been shown in various clinical trials and meta-analyses [26]. Mometasone and clobetasol were commonly prescribed topical corticosteroids, which were found to be more cost-effective. Overall, we have found that utilization of cost-effective and cheaper drugs like prednisolone, betamethasone, mometasone, and clobetasol was significantly more compared to costly alternatives like methotrexate, azathioprine, cyclosporine, and fluticasone (Table 2-4). Prednisolone was also found to be over-utilized as per the PDD/DDD ratio. The current evidence also shows that there is a relative over-utilization of these drugs in skin disorders [7,20].

Over-utilization of cheaper drugs could also be associated with higher incidence and reporting of adverse effects. So, pharmacovigilance activity should also incorporate data from drug utilization studies and pharmacoeconomic surveys in its analysis and this field needs further research and investigation.

The limitation of our study was that we could not do a safety analysis and its correlation with drug utilization and cost-effectiveness, but the finding of this study does highlight the need for such analysis in future research.

## Conclusions

Immunosuppressants are commonly prescribed in patients with skin disorders, which indicates that over-activity of the immune pathway is a common pathogenesis of many skin disorders. The pharmacoeconomic analysis showed that cost-effective drugs were prescribed more frequently than expensive alternatives in line with the current evidence, which shows that the over-utilization of cheaper corticosteroids were found to be more commonly used compared to other immunosuppressants like cyclosporine, azathioprine, and cyclosporine. However, deflazacort was preferred over cheaper corticosteroids due to its better safety profile. Cost-effectiveness of steroids has the advantage of providing better patients’ adherence to pharmacotherapy but could also lead to long-term adverse effects of steroids. Pharmacovigilance research should also incorporate pharmacoeconomic analysis to determine the relation between these two aspects.
